# QSOX1 Modulates Glioblastoma Cell Proliferation and Migration In Vitro and Invasion In Vivo

**DOI:** 10.3390/cancers16213620

**Published:** 2024-10-26

**Authors:** Reetika Dutt, Colin Thorpe, Deni S. Galileo

**Affiliations:** 1 Department of Chemistry and Biochemistry, University of Delaware, Newark, DE 19716, USA; rdutt@udel.edu (R.D.); cthorpe@udel.edu (C.T.); 2 Department of Biological Sciences, University of Delaware, Newark, DE 19716, USA

**Keywords:** QSOX1, glioblastoma, chick embryo brain, xenograft, shRNA, lentivirus

## Abstract

**Background**: Quiescin Sulfhydryl Oxidase 1 (QSOX1) is an enzyme that catalyzes the oxidation of free thiols to generate disulfide bonds in a variety of proteins, including the cell surface and extracellular matrix. QSOX1 has been reported to be upregulated in a number of cancers, and the overexpression of QSOX1 has been correlated with aggressive cancers and poor patient prognosis. Glioblastoma (GBM) brain cancer has been practically impossible to treat effectively, with cells that rapidly invade normal brain tissue and escape surgery and other treatment. Thus, there is a crucial need to understand the multiple mechanisms that facilitate GBM cell invasion and to determine if QSOX1 is involved. **Methods and Results**: Here, we investigated the function of QSOX1 in human glioblastoma cells using two cell lines derived from T98G cells, whose proliferation, motility, and invasiveness has been shown by us to be dependent on disulfide bond-containing adhesion and receptor proteins, such as L1CAM and the FGFR. We lentivirally introduced shRNA to attenuate the QSOX1 protein expression in one cell line, and a Western blot analysis confirmed the decreased QSOX1 expression. A DNA content/cell cycle analysis using flow cytometry revealed 27% fewer knockdown cells in the S-phase of the cell cycle, indicating a reduced proliferation. A cell motility analysis utilizing our highly quantitative *SuperScratch* time-lapse microscopy assay revealed that knockdown cells migrated more slowly, with a 45% decrease in migration velocity. Motility was partly rescued by the co-culture of knockdown cells with control cells, indicating a paracrine effect. Surprisingly, knockdown cells exhibited increased motility when assayed using a Transwell migration assay. Our novel chick embryo orthotopic xenograft model was used to assess the in vivo invasiveness of knockdown vs. control cells, and tumors developed from both cell types. However, fewer invasive knockdown cells were observed after about a week. **Conclusions**: Our results indicate that an experimental reduction in QSOX1 expression in GBM cells leads to decreased cell proliferation, altered in vitro migration, and decreased in vivo invasion.

## Simple Summary

Glioblastoma (GBM) is the deadliest form of primary brain cancer, and the survival of patients is only about 15 months after diagnosis, even with aggressive treatment. Many cellular factors that are used by GBM cells to divide abnormally and invade brain tissue to result in patient death are unknown. Here, we investigated the potential role of the cellular enzyme QSOX1, which creates specific bonds within proteins, in GBM cell proliferation, migration in a dish, and invasion into brain tissue in our chick embryo brain tumor model system. By experimentally reducing QSOX1 protein production in GBM cells, we found that this reduction resulted in less proliferation, slower migration, and less invasion into brain tissue. These results show the importance of the QSOX1 enzyme in GBM cells in order for them to exhibit their abnormal aggressive behavior that drives this incurable cancer.

## 1. Introduction

Some time ago, a flavin-dependent sulfhydryl oxidase enzyme was isolated from chicken egg white; the enzyme was termed Quiescin Sulfhydryl Oxidase (QSOX) after the human growth factor Quiescin Q6 [1]. Although the enzymological roles of QSOX have been extensively studied by the Thorpe group [2], its potential role in health and disease has not yet been fully elucidated. QSOX participates in oxidative protein folding, which is the rapid generation of disulfide bonds in reduced, unfolded proteins with the reduction of molecular oxygen to hydrogen peroxide.

QSOX is found in metazoans, plants, and protists, but is absent in fungi [1,2]. There are two paralogs of QSOX in humans, QSOX1 and QSOX2, which share a 37% sequence similarity and 68% structural identity [2,3]. In humans, QSOX1 has two mRNA splice variants; a long form QSOX1a which contains a C-terminal transmembrane region and a short form QSOX1b without this feature [4]. QSOX1 is located in the endoplasmic reticulum (ER) and the Golgi apparatus inside the cell [3,5]. QSOX1 is also secreted and found in a wide range of biological fluids [1,2] and within the extracellular matrix [5,6,7,8]. Although its biological roles remain to be elucidated, recent studies have aimed to shed light on the roles of QSOX1 in various medical and disease states.

Recent studies have shown QSOX1 to be upregulated in a number of cancers such as pancreatic [9,10], prostate [11,12], breast [13,14,15], esophageal [16], melanoma [17], lung [18], and glioblastoma (GBM) [19]. The overexpression of QSOX1 has been associated with a higher tumor grade, increased cell proliferation, increased cell invasion, and poor prognosis. In prostate cancer, the downregulation of a tumor suppressor and transcriptional regulator gene NKX3.1 in early tumorigenesis was related to QSOX1 overexpression [11]. A peptide from the C-terminal region of QSOX1 was isolated from the plasma of pancreatic cancer patients which correlated to QSOX1 overexpression in patients with tumors [9]. Similarly, in breast cancer, a high expression of QSOX1 was related to poor prognosis and increased invasiveness [14]. In that same study, it was shown that suppressing QSOX1 expression led to significantly decreased proliferation, invasion, and decreased extracellular matrix metalloproteinase (MMP) activity [14]. In glioblastoma, QSOX1 recently was found to be involved in cell viability, cell motility, and tumor size using established GBM cell lines [19]. 

GBM is the most aggressive and devastating type of glioma brain tumor [20,21,22]. Overall, 60% of all primary brain tumors diagnosed each year are gliomas, which arise from the supporting glial cells of the brain or their precursors [23]. GBM tumors also contain stem cells [24,25,26], which increases the complexity of both their pathophysiology and treatment. Gliomas, whether malignant or benign, are dangerous due to their location in the brain [23]. Symptoms of these tumors arise late in patients, thereby leading to late detection, increased tumor progression, and, thus, poor prognosis [27]. Grade IV glioma (glioblastoma; GBM) is the highest-grade glioma recognized by the World Health Organization (WHO) based on cell morphology, malignancy, and pathogenicity, and makes up 15% of all brain tumors [23]. They are characterized by their cell differentiation, high invasiveness, and high malignancy [23,27,28]. GBM is often diagnosed late as the patient already experiences headaches, seizures, memory loss, and vision changes. Due to the late diagnosis and the highly invasive nature of GBM cells, the mean survival time of GBM patients is only about 15 months with aggressive treatment [29]. Surgery, radiation, and chemotherapy are the current treatments, but they essentially are ineffective [27,29].

Although QSOX1 has been found to be upregulated in GBM and GBM-derived cell lines [19], those experimental in vivo studies were performed by the subcutaneous injection of cells, which is not their native location (i.e., the brain). Thus, we investigated the roles of QSOX1 in GBM cell behavior in vitro and in vivo using our embryonic chick brain orthotopic tumor model [30] and have implicated its importance in the invasion of brain tissue. Our chick embryo brain tumor model has proven to be a suitable and useful alternative to rodents for identifying the invasive behaviors of GBM cells and the molecules that control them [30,31,32,33]. It is of the utmost importance to find new molecular factors in GBM that can be used as therapeutic targets, given that GBM currently is incurable. Since QSOX1 is a key factor that drives cell proliferation, invasion, and poor prognosis in a variety of aggressive cancers [9,11,14], QSOX1 could play a similar important role in GBM tumorigenesis and cell invasion into human brain tissue. Here, we provide further evidence of the involvement of QSOX1 in GBM cell pathogenesis.

## 2. Materials and Methods

### 2.1. Cell Lines

Human cell lines used in this study were obtained from American Type Culture Collection (ATCC, Manassas, VA, USA). T98G (CRL-1690) is a human GBM cell line [34]. HEK 293T (CRL-3216) is a human embryonic kidney cell line [35]. Cells were cultured in Dulbecco’s Modified Eagle Medium (Mediatech, Manassas, VA; DMEM; high glucose) supplemented with 10% Fetal Bovine Serum (Gemini Bio-Products, West Sacramento, CA, USA; FBS), 1 mM L-glutamine, and 2 mM penicillin-streptomycin (referred to as complete media) and incubated in a humidified chamber at 37 °C and 5% CO_2_.

### 2.2. Vectors and Transfections

Five different unconfirmed TRC QSOX1 targeting shRNA (short hairpin RNA) expressing lentiviral vectors were purchased from Dharmacon (Lafayette, CO, USA; Accession #: NM_001004128, NM_002826; Cat. #: RHS4533-EG5768). The backbone vector is pLKO.1, which contains a human U6 promoter, and a puromycin selective marker into which a hairpin RNA targeting sequence was inserted. After assessing the set of the vectors for their ability to attenuate QSOX1 expression in human T98G cells, one vector (Clone ID: TRCN0000064186) with the QSOX1 mature antisense sequence ATTTCTCGCAAAGAATCCATC successfully attenuated QSOX1 expression and still allowed cells to grow in culture (see Results). T98G cells with the QSOX1 vector are called T98G/sh86. The control vector was the pLKO.1 vector (Plasmid 10878:pLKO.1-TRC cloning vector, Addgene Inc., Cambridge, MA, USA) without the hairpin RNA insert [31]. 

### 2.3. Lentiviral Transductions

Lentiviral production and transduction followed a previously described method [36]. Briefly, HEK 293T cells were grown to approximately 70% confluence and then transfected with lentiviral vector plasmids (pLKO.1 or sh86), helper plasmid (pCMVΔR8.2; gift from Didier Trono, Salk Institute, San Diego, CA, USA), and the envelop plasmid (pMD.G; gift from Didier Trono) in the proportion of 4:3:1 in a 6-well plate. Transfection was by Lipofectamine 2000 (Invitrogen, Carlsbad, CA, USA), slightly modified from the manufacturer’s protocol. Cell culture media was changed 24 h after transfection and cells were allowed to recover. The media containing viral particles were collected 48 h and 72 h after transfection. The supernatant was collected and filtered through a 0.45 μm filter. Then, 1 mL of this packaged viral vector was added to cells to be transduced along with 10 μg/mL of polybrene (Sigma, St. Louis, MO, USA). Cells were expanded and then selected with puromycin (Sigma) at a concentration of 1 μg/mL for 6 days. Thus, surviving cells were stably transduced with the pLKO.1 or sh86 vectors.

### 2.4. Western Blotting

Cells grown on culture dishes were rinsed three times with PBS and then solubilized with RIPA buffer (Thermo Fisher Scientific, Waltham, MA, USA) containing Complete Mini Protease Inhibitor (Roche Diagnostics GmbH, Mannheim, Germany) over ice for 3 to 5 min. The cells were scraped and solubilized by sonication. The cell supernatant was used to determine protein concentration using the Bradford Assay. Samples were prepared in reducing Laemmli buffer and denaturing conditions. Equal amounts of proteins (20 to 40 μg) were loaded into each well of a 12% SDS polyacrylamide gel along with Dual Color Precision Plus Protein Prestained Standard (Bio-Rad Laboratories, Hercules, CA, USA). The gel was used with Tris-Glycine running buffer at 120 V.

Separated proteins from the gel were transferred onto a PVDF membrane overnight at 4 °C and 30 V. The membrane was blocked with 5% non-fat milk in Tris-Buffered Saline (TBS). Primary rabbit polyclonal QSOX1 antibody (#12713-1-AP, Proteintech, Rosemont, IL, USA) was used at a dilution of 1:500 in 1% non-fat milk in TBS and incubated overnight at 4 °C. The membrane was washed three times with TBS and 0.01% Tween-20 (Sigma) for 5 min each and incubated with HRP-conjugated anti-rabbit secondary antibody (#7074, Cell Signaling Technology, Danvers, MA, USA) for 1 h at room temperature. Blots were washed with TBS/Tween-20 and then incubated with ECL substrate (Thermo Fisher Scientific) and developed and imaged in a FluorChemQ imager (Alpha Innotech, San Leandro, CA, USA). To stain for the loading control GAPDH, the QSOX1 blot was stripped of antibodies and incubated in anti-GAPDH primary antibody (Santa Cruz Biotechnology, Dallas, TX, USA; sc-47724), followed by HRP-conjugated anti-mouse secondary (Jackson ImmunoResearch, West Grove, PA, USA). 

### 2.5. DNA Content and Cell Cycle Analysis

Cells were grown to approximately 50% confluence, rinsed with PBS, and trypsinized with 0.05% trypsin/EDTA [37]. The cells were resuspended in complete media (DMEM with 10% FBS) with 0.003% DNase I (Sigma) and centrifuged at 800 RPM. The cell pellets were then resuspended in 0.5 mL PBS and 4.5 mL 70% ethanol. Cells then were kept at −20 °C for 4 to 5 days for fixation and permeabilization. On the day of DNA staining and flow cytometry analysis, cells were centrifuged and the fixing solution was removed. Cell pellets were resuspended in 0.5 mL cell staining solution containing 200 μg/mL RNase A (Sigma) and 12.5 μg/mL of propidium iodide (Sigma) in PBS. The cells were incubated at room temperature in the dark for 30 min and then transferred to a filter cap tube for analysis with a FACSCalibur flow cytometer (Becton Dickinson, Franklin Lakes, NJ, USA) using 488 nm excitation and FL2 area as the collected data parameter. No compensation was required. Approximately 50,000 cells were analyzed for DNA content, and the percentage of cells in different cell cycle stages was determined using ModFit LT^TM^ software (ver. 3.1 with Service Pack 3; Verity Software House, Topsham, ME, USA) as was previously carried out [36,37].

### 2.6. Fluorescently Labeling Cells

Cells were labeled with Vybrant DiI (V22885, Thermo Fisher Scientific) for *Mixed SuperScratch* Assay and Vybrant DiO (V22886, Thermo Fisher Scientific) for microinjection experiments. Labeling procedure was modified from the manufacturer’s protocol. Cells were trypsinized and resuspended in 1 mL of serum-free media. Then, 5 μL of the dye stock was added to the cells, mixed gently, and incubated at 37 °C for 30 min. The dye was removed by centrifugation and washed in serum-free media. The cells were finally resuspended in warm complete media.

### 2.7. SuperScratch Assay

Cell motility of T98G/pLKO.1 and T98G/sh86 cells were assessed using our highly quantitative *SuperScratch* assay as described previously [32,33,36,37,38]. Cells were grown to confluence in a 6-well plate in complete media. A scratch was made with a P1000 micropipet tip on the cell monolayer. The monolayer was rinsed with PBS, and media with 0.5% FBS (low-serum media) were added. The plate was sealed with tape to prevent evaporation and placed in a culture chamber on an adjustable ProScan II automated stage (Prior Scientific, Rockland, MA, USA) on a Nikon TE2000-E automated microscope (Nikon Instruments, Melville, NY, USA). The chamber was kept at atmospheric conditions of 5% CO_2_ using a gas injection controller (Forma Scientific, Marietta, OH, USA). The temperature was maintained at 37 °C with an air temperature controller (Air Therm, World Precision Instruments, Sarasota, FL, USA) and a temperature-controlled stage insert (Tokai Hit, Shizuoka-ken, Japan). Phase-contrast images were captured every 10 min for 20 h at designated fields of view on the scratch edge using a CoolSnap ES CCD camera (Photometrics, Tucson, AZ, USA) and a 20× Nikon Plan Fluor ELWD objective. MetaMorph Premier Software (ver. 7.6.0.0; Molecular Devices Corporation, Downingtown, PA, USA) was used to operate the time-lapse system.

### 2.8. SuperScratch Mixed Cell Assay

This experiment was conducted to see if the velocity of the QSOX1 knockdown T98G/sh86 cells increased in the presence of neighboring QSOX1-expressing T98G/pLKO.1 cells by a paracrine mechanism (i.e., knockdown cells in close proximity to normal cells), since QSOX1 is known to be secreted. T98G/sh86 cells were labeled with Vybrant DiI as described above. The T98G/sh86/DiI cells were mixed with unlabeled T98G/sh86 or T98G/pLKO.1 at a ratio of 25% to 75%. These mixtures were seeded in a 24-well plate and cultured overnight in complete media. The monolayers were scratched, rinsed with PBS, and supplemented with low-serum media the next day. The plate was then sealed and placed on the 37 °C stage in the presence of 5% CO_2_. Phase-contrast and fluorescent images were captured every 10 min over 20 h at designated fields as stated above.

### 2.9. Cell Motility Analysis

Quantitative analysis of cell motility was performed as described in [36,37,38] using the Track Points function in MetaMorph Premier software (Molecular Devices). First, 10 cells per field of view were analyzed by tracking the movement of their nuclei from the scratch edge as the cells migrated into the denuded scratch area. A Microsoft Excel spreadsheet was generated by MetaMorph which contained the velocities, distances, and times of the individually tracked cells. The overall average velocity of the tracked cells was determined and then converted from pixels/min to microns/min. Time-lapse movies made from the file of successive images collected every 10 min for 20 h are presented in Supplemental Data.

### 2.10. Transwell Migration Assay

Transwell migration assays were performed as described in [39] with slight modifications. Briefly, T98G/pLKO.1 and T98G/sh86 cells were trypsinized, collected from the dish in complete media, pelleted, and resuspended in serum-free DMEM at a concentration of 4.5 × 10^5^ cells/mL. Then, 100 μL of a cell suspension was added to an 8 μm pore size Transwell insert (#3422, Corning, Kennebunk, ME, USA). Then, 750 μL of complete media was added to the wells of a 24-well plate, and the Transwell inserts with cell suspension were lowered into them and placed in the CO_2_ incubator. After 20–24 h at 37 °C, the cells on the upper membrane of the inserts were removed with a cotton swab. The cells that had migrated to the underside of the inserts were fixed in methanol for 10 min and then stained with crystal violet solution for 10 min. The number of migrated cells were observed and counted under the microscope from 4 different fields of view. 

### 2.11. Chick Embryo Brain Microinjections

The cells used in the microinjections were T98G/pLKO.1 and knockdown T98G/sh86 labeled with Vybrant DiO as described above. Cells were trypsinized and resuspended in complete DMEM with an additional 30% Matrigel Matrix (Corning) added to the cell suspension. Matrigel has been shown to be necessary for T98G tumor engraftment in mice and chick embryo brains [31,40]. The cell density for injection was 10,000 cells/μL before adding the additional Matrigel.

Fertile White Leghorn chicken embryos were obtained from the University of Delaware Department of Animal and Food Sciences. The eggs were incubated starting on embryonic day (E) 0 until embryonic day 5 (E5) in a humidified incubator at 37.5 °C. Embryonic chick optic tecta (OT; midbrains) were injected with fluorescently labeled GBM cells. The experimental procedure was detailed previously [30,32]. Briefly, the eggs were cleaned with 70% ethanol and candled, and a small window was cut over the air space at the top of the eggs. Then, 5 μL of cell suspension containing approximately 50,000 cells were microinjected into the OT using a PV830 pneumatic picopump (World Precision Instruments; Sarasota, FL, USA). After injection, a few drops of sterile 50 mg/mL ampicillin were added over the embryo, the window in the shell was sealed with transparent tape, and the eggs were placed back in the incubator until E10 or E13.

At E10 or E13, the embryos were sacrificed, and the brains were dissected. The OTs were fixed in 2% paraformaldehyde in 0.1 M cacodylate buffer (Electron Microscopy Sciences, Hatfield, PA, USA) and then embedded in 3.5% agar and 8% sucrose in PBS and sectioned at 350 μm using a Vibratome (model 3000). The sections were then screened for green fluorescent tumors using a Nikon SMZ1500 stereo microscope equipped with epifluorescence. Sections containing fluorescent tumors then were mounted on slides for observation using a Nikon E800 epifluorescence microscope connected to a Nikon C1 confocal microscope with a 488 nm argon laser. Images of vibratome sections were collected as z-stacks and presented as maximum projections using Nikon EZC1 software (ver. 2.00).

### 2.12. Statistical Analysis

The data are presented as mean ± standard error of the mean of at least three repeats. Unpaired Student’s *t*-tests were used to determine significance between data. A *p*-value ≤ 0.05 was considered to be statistically significant.

## 3. Results

### 3.1. shRNA Vector Decreased QSOX1 Expression in T98G/sh86 Cells

**Figure 1:**
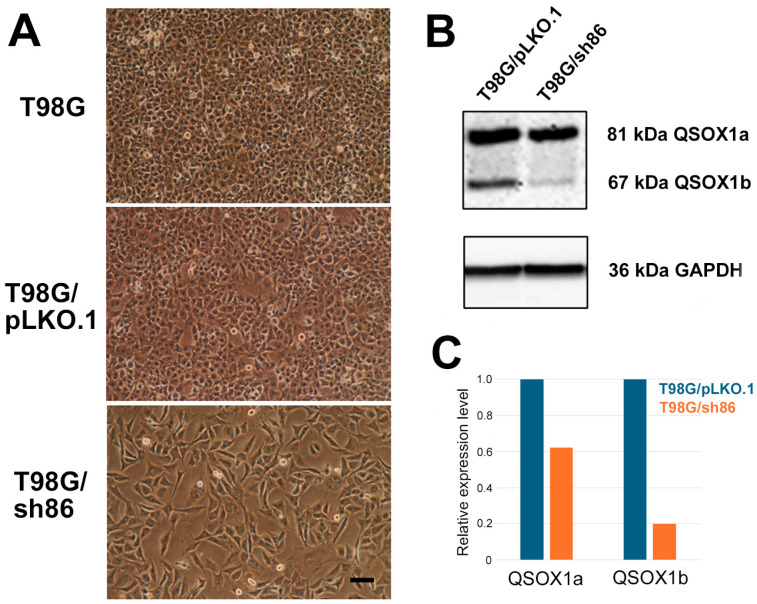
Cell appearance and Western blot analysis. (**A**) Phase-contrast images of uninfected T98G, control T98G/pLKO.1, and QSOX1 knockdown T98G/sh86 cells. Bar, 100 μm. (**B**) Western blot analysis of T98G/pLKO.1 and T98G/sh86 cell lysates. Human anti-QSOX1 antibody was used for probing QSOX1 expression and anti-GAPDH antibody was used as loading control. (**C**) Quantitation of Western blot showing relative expression levels of QSOX1a and QSOX1b in T98G/pLKO.1 and T98G/sh86 cells. Expression levels are normalized to GAPDH levels. The original Western blot figure can be found in Supplementary Figure S1.

Derivatives of Grade IV human GBM cell line T98G were used for our experiments, since we previously used T98G-derived cells to demonstrate the role of L1CAM in cell motility and brain invasiveness in multiple studies in vitro and in vivo [31,33,36,37]. Since L1CAM contains six Ig-like domains, and, since FGFRs that facilitate L1CAM signaling in T98G cells [36] also contain three Ig-like domains, all of which contain disulfide bonds, these cells seemed ideal for investigating the potential role of QSOX1 in GBM cell proliferation, motility, and invasion. T98G cells are tile-like in appearance in vitro, are rapidly growing, and are very invasive in our chick embryo brain tumor model. The Western blot analysis of these cells showed the presence of both QSOX1a long form (81 kDa) and QSOX1b short form (67 kDa) (Figure 1). Lentiviral shRNA was used successfully to knockdown the QSOX1 expression in these cells. Five different shRNA constructs were screened to determine the most effective, viable knockdown. The TRCN0000064186-construct-infected cells, termed T98G/sh86, not only survived, they withstood cell passaging and showed a decrease in QSOX1 expression. Thus, these cells were used for further experimentation. Cells infected with the four other constructs exhibited characteristics such as slow growth, low survivability, cell death over time, and susceptibility to cell passaging, which made them unsuitable for further experimentation. The lack of survival of these other four cell lines potentially was due to the excessive attenuation of QSOX1, which resulted in them being unviable, but we were not able to confirm this. The pLKO.1 vector was used to produce control T98G/pLKO.1 cells. The T98G/pLKO.1 cells also looked similar to the uninfected T98G cells; they formed a tile-like monolayer of cells in a dish. However, the morphology of the T98G/sh86 cells was altered. They appeared spindle-shaped, with spaces in between each other, and did not form a tight tile-like monolayer like control cells (Figure 1A).

The Western blot analysis of T98G/sh86 showed that the QSOX1 expression was decreased compared to the T98G/pLKO.1 controls (Figure 1B). The QSOX1b short form was decreased by 80%, and the long form QSOX1a was decreased by approximately 40% (Figure 1C and Supplementary Figure S1). This level of reduction in QSOX1 was sufficient to cause phenotypic abnormalities, yet presumably not sufficient to result in the cells becoming unviable. The T98G/pLKO.1 QSOX1 expression was similar to that of uninfected T98G cells. 

### 3.2. QSOX1 Knockdown Decreased T98G Cell Proliferation

**Figure 2:**
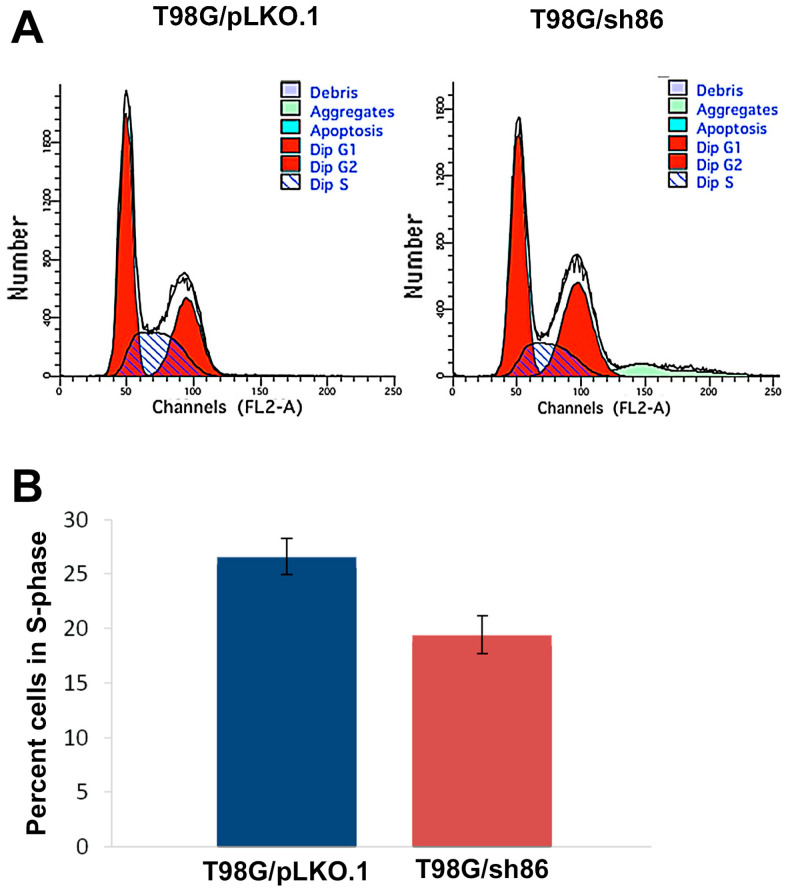
Cell cycle/DNA content analysis of GBM cells. (**A**) Histograms generated by ModFit LT software depicting various stages of the cell cycle in T98G/pLKO.1 and T98G/sh86 cells. S-phase is depicted by the striped region between the red G1 and red G2 peaks and is an indicator of cell proliferation. (**B**) Average percentage of cells in the S-phase for T98G/pLKO.1 and T98G/sh86 cells. In total, 50,000 cells per cell type were analyzed per experiment. Graph depicts data from 3 separate experiments. *p*-value < 0.05.

The doubling time of T98G/pLKO.1 cells was similar to that of uninfected T98G, with it being around every 24 h. However, the T98G/sh86 cells grew at a much slower rate in culture, hardly ever reaching complete confluency. Cell proliferation was studied using a cell cycle/ DNA content analysis with the flow cytometer. Cells were fixed, and DNA was stained with propidium iodide. In total, 50,000 cells per cell type (T98G/sh86 or T98G/pLKO.1) were analyzed. The percentage of cells in the S-phase of the cell cycle is an indicator of the extent of cell proliferation. Our analyses showed that 26% of T98G/pLKO.1 cells were in the S-phase, while only 19% of T98G/sh86 cells were in the S-phase (Figure 2A,B). These results indicated that the number of T98G/sh86 cells proliferating were measurably less than T98G/pLKO.1 cells. Thus, QSOX1 attenuation in GBM cells led to decreased cell proliferation. We did not detect degraded DNA levels (i.e., apoptosis) in control or knockdown cells, which would have been evident as DNA content to the left of the G1 peak in our DNA content graphs and detected by the analysis software. Thus, cell death did not appear to be occurring in either of our cultures and was not pursued further.

### 3.3. QSOX1 Knockdown Decreased T98G Cell Motility In Vitro

**Figure 3:**
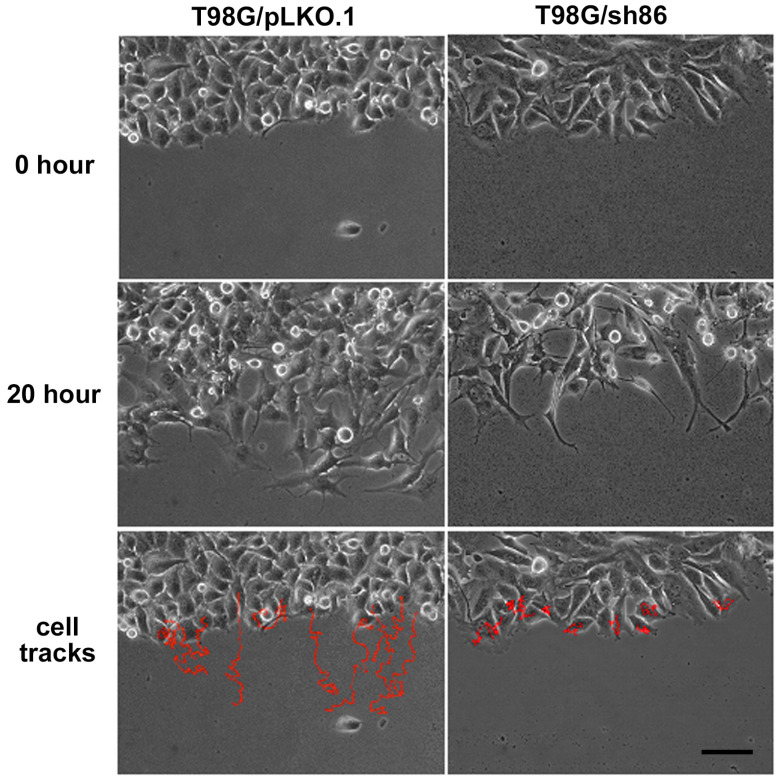
*SuperScratch* Assay images for cell motility. Phase-contrast images of T98G/pLKO.1 (**left**) and T98G/sh86 (**right**) cells at the start (0 h) and at the end (20 h). Bottom cell tracks row shows paths taken by tracked cells in red superimposed on 0 h images. Bar, 100 μm.

**Figure 4:**
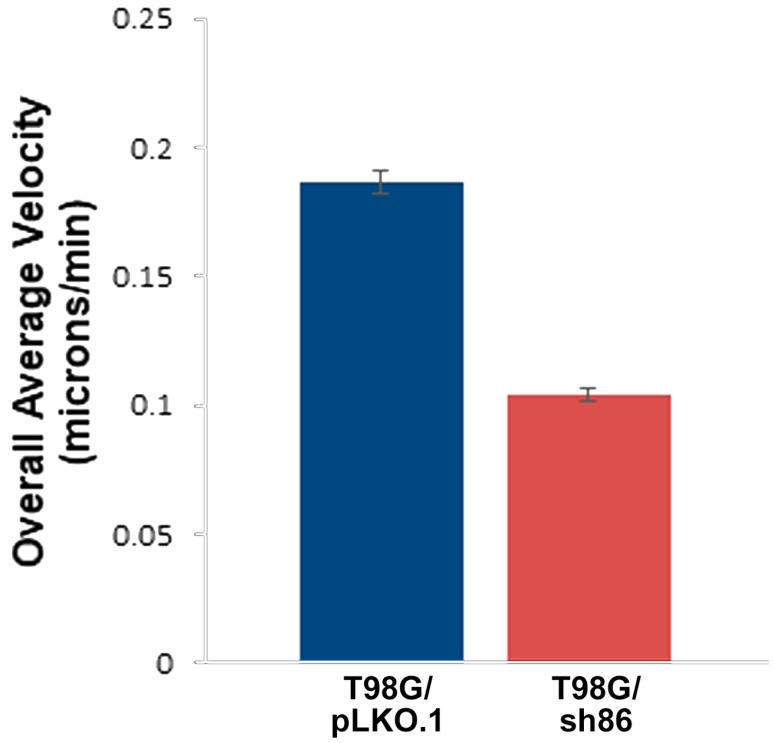
*SuperScratch* Assay measurement of cell velocity. Graph shows the overall average velocity of cells (microns/minute) over the 20 h period. In total, 100 total cells per cell type were analyzed from 3 separate experiments (10 cells/field of view). *p*-value < 0.001.

GBM cells are characteristically highly motile and can migrate rapidly into cleared spaces in vitro. We used our *SuperScratch* assay to determine if cell motility was affected by QSOX1 attenuation. Cells were grown to 90% confluence and a scratch was made to provide a cleared space into which the cells migrated. Fields of view were selected and photographed every 10 min for over 20 h to determine the number and distance of cells that had moved. Cell movement was measured by tracking the movement of the nuclei over time, as this was a consistently visible and unambiguous “marker” within each cell. Our analyses showed that T98G/pLKO.1 cells migrated into the scratched space more rapidly than did the T98G/sh86 cells. The overall pathway and distance moved by the cells are shown in Figure 3 by the red track lines. The T98G/sh86 cell track lines were shorter than those of the T98G/pLKO.1 cells, indicating that the knockdown cells migrated less distance than the controls over the time course of the experiments. In total, 100 cells per cell type were quantitatively analyzed from three different experiments. Refer to Supplementary Videos S1 and S2 for time-lapse movies of these experiments. Figure 4 shows the graph based on the velocity of the cells as determined by the MetaMorph software, which reveals that motility was reduced in QSOX1-attenuated T98G/sh86 cells by approximately 45%, compared to controls.

### 3.4. Decreased Cell Motility Was Partially Rescued by Co-Culture with Control Cells

**Figure 5:**
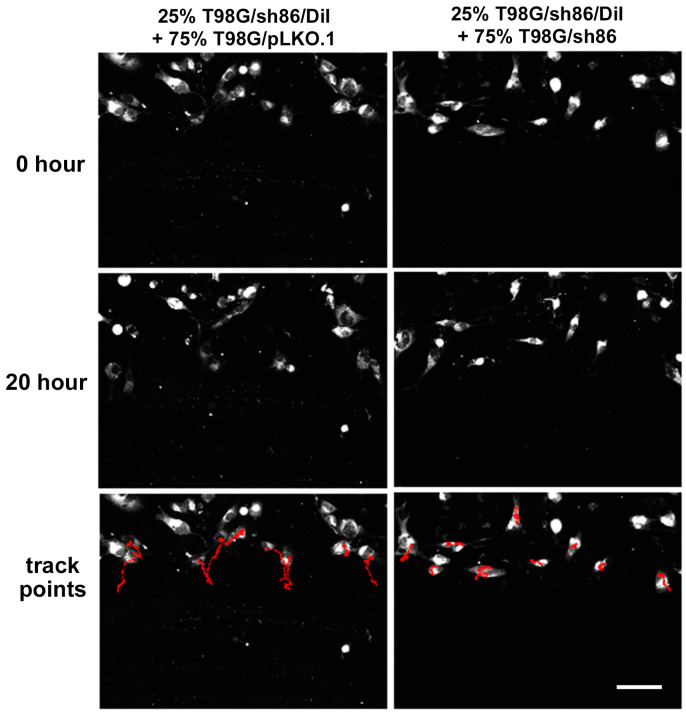
*SuperScratch* Assay for paracrine effect. Fluorescence images of co-culture experiments of 25% T98G/sh86/DiI + 75% T98G/pLKO.1 (**left**) and 25% T98G/sh86/DiI + 75% T98G/sh86 (**right**) at the start (0 h) and at the end (20 h) of time-lapse image collection. T98G/sh86/DiI cells appear as white. Track points row shows paths traveled by tracked cells over the course of the experiment in red superimposed on 0 h images. Bar, 100 μm.

**Figure 6:**
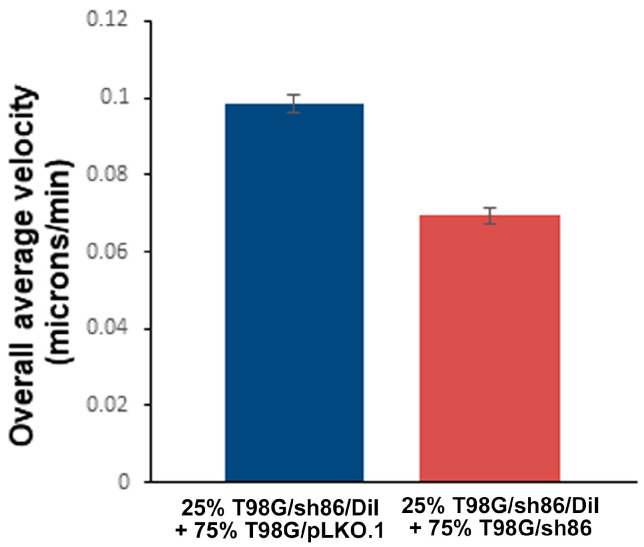
*SuperScratch* Assay measurement of paracrine effect. Graph showing the overall average velocity of cells (microns/minute) over the 20 h period. In total, 60–90 total cells per cell type were analyzed from 3 separate experiments (10 cells/field of view). *p*-value < 0.001.

QSOX1 is found not only in the plasma membrane but is also secreted extracellularly (e.g., into cell culture media), where it potentially could influence nearby cell behavior. Therefore, we investigated the potential effect of paracrine QSOX1 secreted by control cells on the motility of QSOX1 knockdown T98G/sh86 cells using a *SuperScratch* assay where knockdown cells were co-cultured with control cells. Fluorescently labeled T98G/sh86 cells (T98G/sh86/DiI) were mixed with unlabeled T98G/pLKO.1 or T98G/sh86 cells in a 25% and 75% ratio, respectively. The T98G/sh86/DiI cells then were tracked along the scratch edge every 10 min over 20 h by virtue of their fluorescence. The T98G/sh86/DiI cells in the presence of T98G/pLKO.1 cells moved farther into the scratch area as shown by the red track lines (Figure 5), indicating the rescue of migration by paracrine QSOX. However, the red track lines of the T98G/sh86/DiI in the presence of T98G/sh86 were much shorter, indicating much reduced migration (i.e., no rescue). In total, 60–90 cells per cell type were analyzed from three different experiments. Refer to Supplementary Videos S3–S6 for time-lapse movies of these experiments (fluorescent and phase-contrast). This analysis revealed that the velocity of T98G/sh86/DiI cells partially and significantly increased (i.e., was partially rescued) in the presence of T98G/pLKO.1 cells (Figure 6). Thus, this study showed that T98G/pLKO.1 cells exerted a paracrine effect on the T98G/sh86/DiI cells that increased their motility.

### 3.5. QSOX1 Knockdown Increased Transwell Migration Ability of T98G Cells

**Figure 7:**
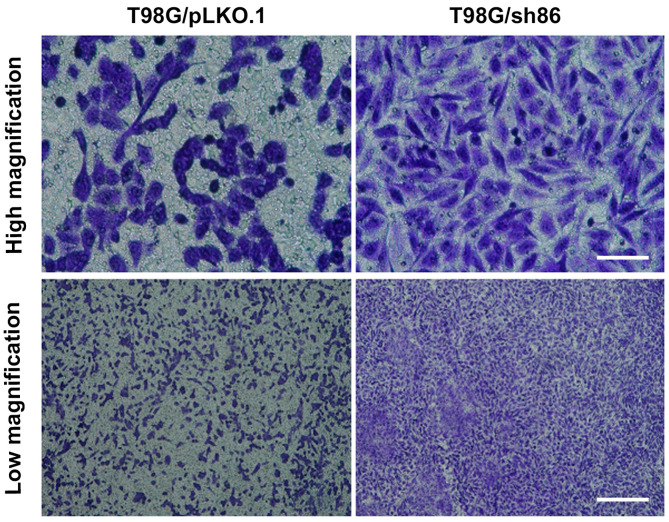
Transwell cell migration. Light micrographs of migrated T98G pLKO.1 and T98G sh86 stained with crystal violet and visualized on the underside of Transwell inserts at end of experiment. Bars: high magnification, 100 μm; low magnification, 500 μm.

**Figure 8:**
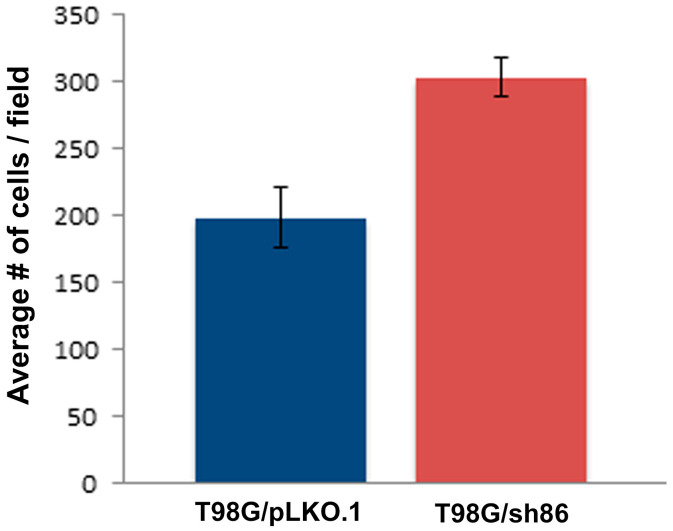
Quantitation of Transwell cell migration. Graph showing the number of migrated cells per field of view on the underside of the Transwell inserts. Cells were counted from 24 fields of view per cell type (2 separate experiments × 3 replicate Transwell filters per experiment × 4 fields of view per Transwell filter). *p*-value < 0.05.

Another experiment commonly used to study cell motility is the use of a Transwell migration assay. T98G/sh86 or T98G/pLKO.1 cells were seeded in the upper chamber of the Transwell insert and allowed to migrate through 8-micron-diameter membrane pores to the bottom of the membrane over 20 to 24 h. The migrated cells on the bottom of the membrane were stained and counted (Figure 7). A chemotactic attractant (10% FBS) was contained in the bottom media to aid in this chemotactic movement. Four fields of view per Transwell were analyzed, and experiments were conducted in triplicate. Surprisingly, approximately 50% more T98G/sh86 cells migrated through the pores to the bottom of the membrane compared to T98G/pLKO.1 control cells (Figure 8). 

### 3.6. QSOX1 Knockdown Decreased Invasion of T98G Cells In Vivo

**Figure 9:**
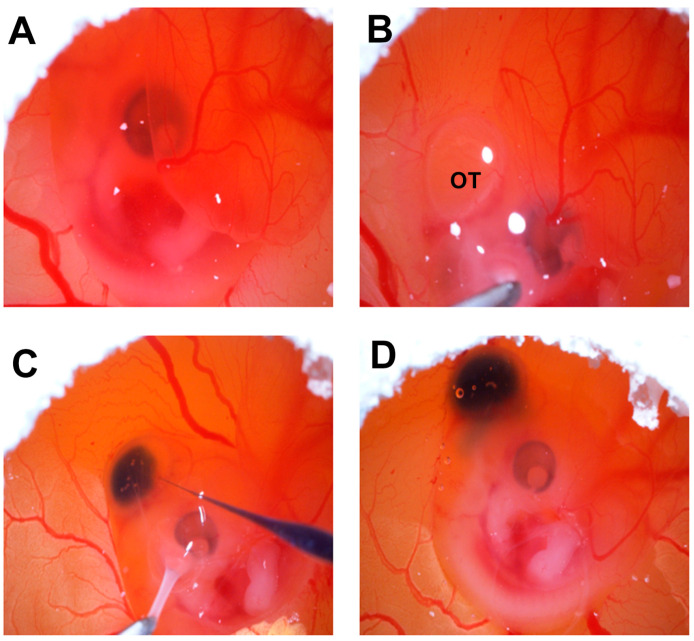
Embryonic chick brain microinjection. Images acquired through dissecting microscope during microinjection process. (**A**) Embryonic day E5 chick embryo inside shell viewed through a hole cut at the top of the egg. (**B**) E5 embryo being held by its amnion membrane showing visible optic tectum (OT). (**C**) Embryo held by amnion membrane with OT being injected with GBM cells mixed with dye. (**D**) E5 embryo immediately after injection of OT with cells showing extent of ventricle.

**Figure 10:**
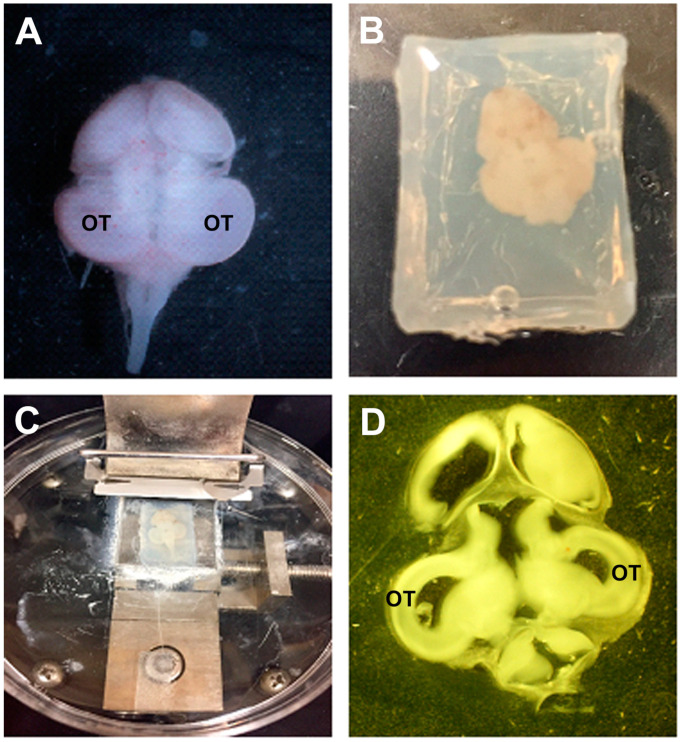
Tissue preparation for visualization. (**A**) E10 whole brain post injection. (**B**) Brain embedded in agar. (**C**) Embedded brain being sectioned on a vibratome. (**D**) Sectioned whole brain slice for mounting and visualization. OT, optic tectum.

**Figure 11:**
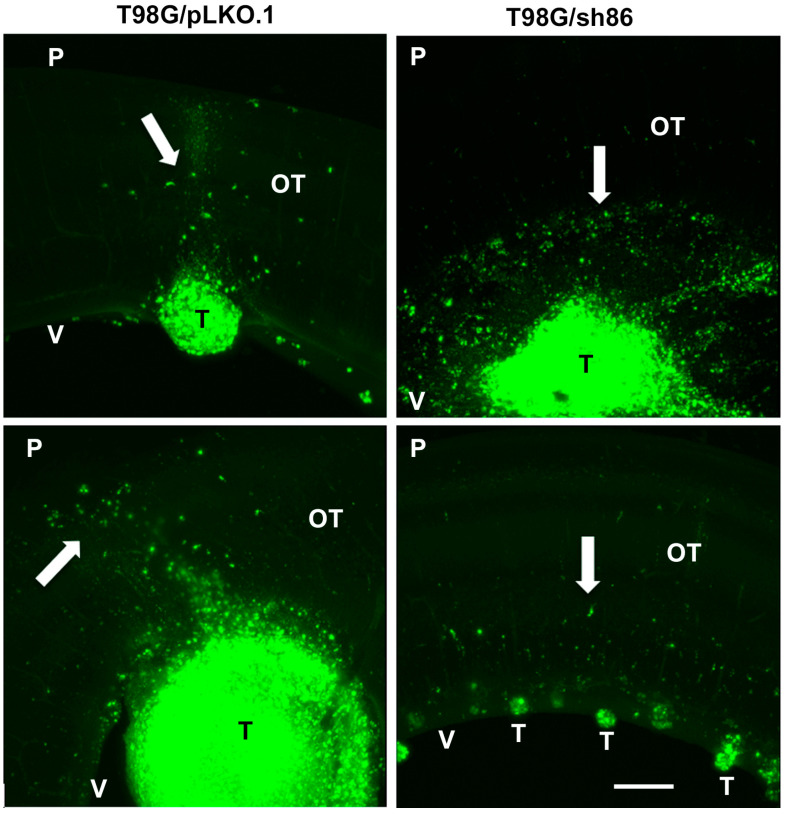
E10 chick brain sections with T98G/pLKO.1 and T98G/sh86 tumors. Invading cells labeled with Vybrant DiO are indicated by white arrows. Images are maximum intensity projections from confocal z-stacks. OT, optic tectum; T, tumor; P, pial surface; V, ventricular surface. Bar, 500 μm.

**Figure 12:**
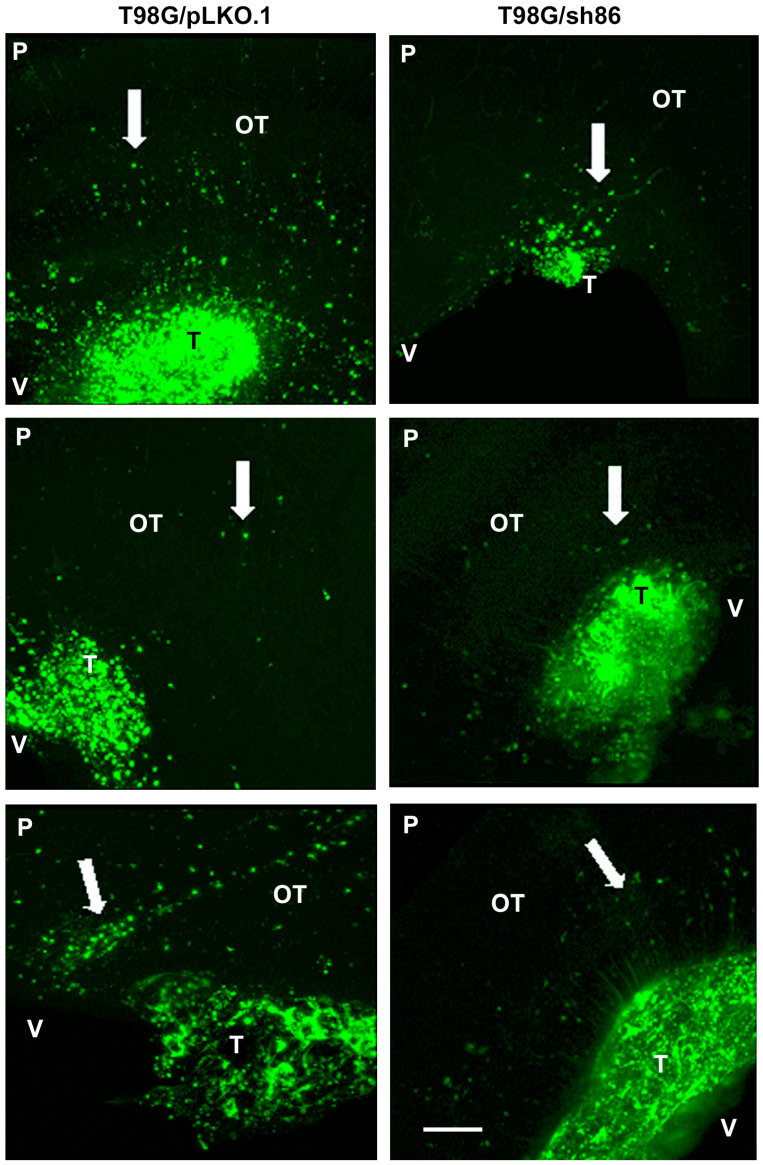
E13 chick brain sections with T98G/pLKO.1 and T98G/sh86 tumors. Invading cells labeled with Vybrant DiO are indicated by white arrows. Images are maximum intensity projections from confocal z-stacks. OT, optic tectum; T, tumor; P, pial surface; V, ventricular surface. Bar, 500 μm.

GBM tumors in patients exhibit highly invasive cells, which is a fundamental reason for the failure of treatment. To determine the role of QSOX1 in GBM cell invasiveness, we used our xenograft chick embryo brain tumor model for these experiments. Fluorescently labeled knockdown T98G/sh86/DiO cells and control T98G/pLKO.1/DiO cells were injected into the optic tectum of embryonic day 5 (E5) chicks as before. The cells were resuspended in high-serum media plus 30% Matrigel (Figure 9) for injection. These cells were allowed to grow into tumors for 5 and 8 days, with the chick embryos sacrificed on E10 and E13. The brains were fixed, thick-sectioned, and imaged. Each brain generated 3 to 5 tumor-containing thick sections (Figure 10). At E10, 10 out of 12 T98G/pLKO.1-injected brains exhibited tumors, but only 2 out of 10 T98G/sh86-injected brains exhibited tumors (Table 1). Tumors from both cell lines showed invasion into the brain tissue; however, T98G/sh86 cells appeared to invade *more* than the T98G/pLKO.1 control cells at E10 (Figure 11). The precise quantitation of invasion was not possible due to the variability of tumor size and extensive invasion of both cell types. At E13, 9 out of 15 T98G/pLKO.1-cell-injected brains exhibited tumors and 7 out of 16 T98G/sh86-cell-injected brains exhibited tumors (Table 1). Both cell lines formed invasive tumors, but, at this later time point, T98G/sh86 cells appeared to invade *less* into the brain than did T98G/pLKO.1 cells (Figure 12). Knockdown T98G/sh86 cell invasion appeared to decrease from E10 to E13, while control T98G/pLKO.1 cell invasion appeared to increase from E10 to E13. Therefore, the QSOX1 knockdown ultimately decreased T98G cell invasion in vivo after an initial increase. However, as stated above, precise quantitation was impossible because tumors formed differently and randomly in individual embryos, and it was not possible to count all the cells that invaded beyond the tumor margin.

## 4. Discussion

Glioblastoma (GBM) is the most highly aggressive, malignant, and invasive primary tumor of the brain [20]. Despite surgery, radiation, and chemotherapy, GBM almost invariably recurs, and the prognosis is very poor in patients. It is very important to understand the multiple molecular mechanisms that contribute to GBM progression, since they may lead to potential therapeutic targets [41,42]. Quiescin Sulfhydryl Oxidase 1 (QSOX1), a flavo-enzyme involved in oxidative protein folding, has been correlated with cell invasion, proliferation, and poor prognosis in pancreatic [9,10], prostate [11,12], and breast [13,14,15,43] cancers. This motivated us to investigate the potential role(s) of QSOX1 in GBM. We showed, using T98G-derived GBM cell lines, that lentiviral QSOX1 knockdown (1) decreased cell proliferation in vitro, (2) decreased cell motility in vitro in monolayer cultures, (3) increased cell migration in a Transwell assay, and (4) ultimately decreased cell invasion into brain tissue in vivo. We also showed that the decreased cell motility of knockdown cells in vitro could be partially rescued by a co-culture with control cells, likely by a paracrine mechanism. These results show the importance of this enzyme in major functions of GBM cells, presumably by inhibiting the formation of disulfide bonds in proteins necessary for those functions. 

Others [19] have investigated the role of QSOX1 in GBM cell viability, migration, and invasion through Matrigel, presumably using the U-87 MG, U-343 MG, and U-251 MG GBM cell lines and other cells (although they apparently misnamed those cell lines in their report as “U87,” “U343,” and “U251” cells). They found that the lentiviral knockdown of QSOX1 decreased cell viability, but they did not strictly examine cell *proliferation* as we did here using a DNA content and cell cycle analysis via flow cytometry. They also found that the QSOX1 knockdown decreased GBM tumor size in mice. However, they injected cells subcutaneously and not orthotopically as we did here, so their in vivo results are difficult to extrapolate to what might occur in the brain. A “wound healing” assay was performed to assay cell motility, but that assay is generally based on assumptions that we demonstrated might not be true [38], and the results are much less accurate than our *SuperScratch* assay that collects data on a single-cell basis before averaging the results. Furthermore, their “invasion” assay was on membrane inserts in vitro through a layer of Matrigel, which primarily is composed of laminin. Laminin is not present in any significant quantity in the brain parenchyma through which GBM invasion occurs. It is a component of basement membranes surrounding blood vessels, on which GBM cells migrate but do not typically invade through (i.e., GBM does not typically metastasize). Thus, we are the first to show that lentiviral QSOX1 knockdown in GBM cells strictly decreased cell proliferation in vitro, decreased individual cell motility (velocity) in vitro, and ultimately decreased invasion into brain tissue in vivo. 

Derivatives of the Grade IV human GBM cell line, T98G [31,34], were used for our experiments. T98G was transduced with a QSOX1 knockdown shRNA containing a lentiviral vector. The transduced cells were selected with puromycin to generate QSOX1 knockdown T98G/sh86 cells. Control T98G/pLKO.1 cells were generated using the “empty” pLKO.1 vector. The morphologies of the uninfected T98G and T98G/pLKO.1 controls were similar, tile-like, and formed a compact monolayer. However, the T98G/sh86 knockdown cells appeared more spindle-shaped (Figure 1A). A Western blot analysis of the lysates of T98G/pLKO.1 and T98G/sh86 cells showed a substantial decrease in QSOX1 expression in T98G/sh86 as expected (Figure 1B). Both forms of QSOX1, QSOX1a and QSOX1b, were detected. Although the shRNA targeted a sequence common to both forms, it is interesting to note that QSOX1b appeared to be more attenuated than QSOX1a. Since, QSOX1a contains a transmembrane region, it potentially could accumulate more stably than QSOX1b. In addition, the QSOX1b could be more susceptible to degradation. Moreover, the message for these two forms might be differentially affected before protein translation. Even though QSOX1b was more attenuated than QSOX1a, differences in cell morphology and cell growth were observed. It is interesting to note that Geng et al. [19] did not describe this issue or which form(s) of QSOX1 was reduced in their knockdown cells. 

The doubling time of the control T98G/pLKO.1 cells was similar to that of the uninfected T98G, being around 24 h. However, T98G/sh86 cells grew much more slowly in culture than T98G/pLKO.1 cells and seldom reached confluence. This decreased cell proliferation was formally studied by analyzing the DNA content by flow cytometry and cell cycle analysis. The percentage of cells in the S-phase is indicative of the percentage of cells in the culture undergoing cell proliferation, and S-phase fraction has been used as an independent prognostic factor of long-term survival in patients with invasive breast carcinoma [44], which underscores the importance of this analysis. It was found that, indeed, fewer cells were proliferating in T98G/sh86 cultures than in T98G/pLKO.1 cell cultures (Figure 2A,B). This decreased cell proliferation as a result of QSOX1 knockdown is in agreement with previous studies on pancreatic [10] and breast [14] cancers.

T98G GBM cells are highly motile cells that have been used extensively in GBM research [45,46,47,48] and form invasive brain tumors in xenograft models [31,33,40]. We previously used T98G cells in our novel chick embryo brain tumor model and in vitro assays to reveal the roles of the adhesion protein L1CAM [49,50] and the fibroblast growth factor receptor (FGFR; a receptor for L1CAM) [31,33,36,37,51], both of which contain multiple disulfide bonds in their Ig-like domains [52,53], in cell motility, cell proliferation, and brain tissue invasion. We used our highly quantitative *SuperScratch* Assay [31,32,33,36,37,38] to determine changes in cell motility by measuring individual cell velocities, which is superior to using the wound healing assay for cell migration [54]. The QSOX1-attenuated T98G/sh86 cells moved much more slowly (approximately half the velocity) than the T98G/pLKO.1 control cells over a 20 h period (Figure 3 and Figure 4). These experiments were conducted in the presence of low-serum media so that the difference in velocities measured were due to QSOX attenuation and not because of the potential stimulatory effects of high-serum media. Thus, the decreased QSOX1 expression led to a decreased cell motility and velocity. Since normal cell motility in T98G cells involves L1CAM and FGFRs [31,33,36,37], both of which contain disulfide bonds in their immunoglobulin domains, it is tempting to hypothesize that the reduced cell velocity of T98G/sh86 cells is due to the disruption of the immunoglobulin domains in these proteins, at least in part. 

The *SuperScratch* Assay also was used to investigate a potential paracrine effect of T98G/pLKO.1 cells on T98G/sh86 cells. T98G/sh86 cells were labeled with Vybrant DiI and their movement was tracked over a 20 h period in the presence of unlabeled T98G/pLKO.1 or T98G/sh86 cells, and their velocities were graphed (Figure 5 and Figure 6). T98G/sh86 cells moved faster in the presence of neighboring T98G/pLKO.1 cells than with T98G/sh86 cells. The rescued, increased velocity of the T98G/sh86 cells was not as high as the control T98G/pLKO.1 cells. However, the increase was still significant compared to the velocity of T98G/sh86 cells. Different percentages of cell mixtures originally were tested, and 25% T98G/sh86 DiI mixed with 75% T98G/pLKO.1 cells was found to be a suitable mixture and was used for our experiments. It is known that QSOX1 is secreted and is available in conditioned cell media [8] and in the ECM [5]. The Fass group has also shown that adding recombinant QSOX1 into cell culture media was able to rescue the adhesion and cell number of QSOX1-attenuated WI-38 (fibroblast) cells [5]. Additionally, it was recently shown that adding exogenous QSOX1b stimulates the migration of a fibroblast cell line and that this effect is regulated through endocytosis by the fibroblasts [55]. Thus, it is likely that QSOX secreted by control T98G/pLKO.1 cells rescued the velocity of QSOX1-attenuated T98G/sh86 cells by a paracrine mechanism. Alternatively, if our hypothesis above is true concerning the requirement for L1CAM for normal motility, then the rescued motility could directly be due to the released “normal” L1CAM either as a soluble ectodomain [31] or as a transmembrane form in exosomal vesicles [33]. 

Our Transwell migration results were unexpected. We previously used this assay to study L1CAM-dependent breast cancer cell migration [39]. In this assay, a chemotactic serum gradient is presented that attracts the cells suspended in low-serum media to pass through 8-micron pores towards high-serum media and has been used by others to assess T98G cell migration [56,57,58]. In this assay, however, we found that an increased number of knockdown T98G/sh86 cells migrated compared to T98G/pLKO.1 control cells (Figure 7 and Figure 8), which was unexpected given the results using breast cancer cells [39] as well as our other assays using T98G cells. We do not know the reason for increased T98G/sh86 migration across the membrane, which seemingly is in contradiction to the *SuperScratch* assay results. A potential factor affecting increased T98G/sh86 migration through the pores could be their altered morphology. T98G/sh86 cells were more spindle-shaped than the tile-like T98G/pLKO.1 cells. This spindle-like morphology could effectively have allowed the cells to traverse more easily through the 8-micron pores to the underside of the Transwell insert. Alternatively, QSOX1 knockdown cells could be more sensitive and reactive to a chemotactic serum gradient after other factors controlling motility (e.g., L1CAM) have been disrupted, or for other unknown reasons. In any case, our results demonstrate that measurements of cell motility/migration can be highly context-specific. 

Another hallmark of cancer is tissue invasion [59]. GBM cells, including T98G, are highly proliferative, motile, and invasive. Since we already have shown that QSOX1 facilitated GBM cell proliferation and motility, it was likely that it also might affect invasion. The overexpression of QSOX1 previously has been associated with highly invasive pancreatic [9,10], breast [13,14,43], and prostate [11,12] cancers. Others have used the chick embryo as an in vivo model for cancer cell growth and invasiveness, but this primarily has been done using the chorioallantoic membrane (CAM) as the site of cell transplantation [60,61,62,63,64]. We used our embryonic chick brain tumor model [30,31,32,33] to form tumors and visualize cell invasiveness into brain tissue. E5 chick embryo midbrains were microinjected with T98G/sh86/DiO and T98G/pLKO.1/DiO cells and were then sacrificed on E10 and E13 (Figure 9 and Figure 10). On E10, T98G/sh86 cells invaded in a similar pattern to the T98G/pLKO.1 cells (Figure 11). However, on E13, T98G/pLKO.1 cell invasion increased compared to the T98G/sh86 cells (Figure 12). Thus, the T98G/pLKO.1 cells exhibited an apparent increased invasion over time, while the QSOX1-attenuated T98G/sh86 cells appeared to exhibit decreased invasion over time. The decreased QSOX1 expression in these cells, thus, may have contributed to the decreased cell invasion, and possibly even decreased cell survival and proliferation over time, which we did not investigate in vivo. Since the injected cells initially were suspended in high-serum media also containing Matrigel, that might explain the similarity of the invasiveness at the earlier analysis time point. However, any such initial potential effect of the serum or Matrigel was not sustained, due to the apparent differences between the invasiveness of the two cell types at the later time point of analysis. Alternatively, the differences in invasion at the different time points may be a reflection of the complex environment in brain tissue that is not present when assaying simple motility in vitro. As is the case for our in vitro motility results, it is tempting to hypothesize that the reduced invasion in vivo was due to the disruption of L1CAM function, which we previously have shown to be important for the invasion of T98G cells in our chick embryo brain tumor model [31,33]. 

Our experiments presented here add to the knowledge that supports an important role for QSOX1 in the progression of cancer and, specifically, GBM cancer. What is still unknown includes the identification of the proteins that are not being processed by QSOX1, which are needed for increased cell proliferation, motility, and invasion. Multiple proteins likely are affected, since these cellular processes are complex and require many proteins with disulfide bonds to act in concert. Two candidate disulfide-bond-containing proteins that could have been affected negatively by QSOX1 attenuation are L1CAM and FGFRs, which we have shown to be necessary for T98G cell motility, proliferation, and invasiveness [31,33,36,37]. The involvement of QSOX1 in cancer progression hopefully will lead to the discovery of key proteins that can be targeted for therapeutic intervention or to targeting the function of QSOX1 itself [65,66]. Future research could include experiments that address whether or not L1CAM and FGFRs, or other cell surface or signaling proteins that contain disulfide bonds, are responsible for the altered behavior of T98G cells in the work described here. 

## 5. Conclusions

QSOX1 protein attenuation significantly decreases proliferation and alters motility in GBM cells compared to when its expression is unattenuated, thus indicating an important role in these cellular processes. QSOX1 also appears to facilitate the invasion of GBM cells in brain tissue; however, this result is less clear due to the difficulty of quantitating invasive behavior in vivo in our model system. 

## Figures and Tables

**Table 1:** **Embryonic chick brain injection data.**

Cell Type Injected	Number of Embryos Injected	Number of Embryos Alive	Age at Dissection	Invasion
T98G/plKO.1/DiO	12	10	E10	Yes
T98G/sh86/DiO	10	2	E10	Yes Similar to controls
T98G/plKO.1/DiO	15	9	E13	Yes
T98G/sh86/DiO	16	7	E13	Yes, but appears less than controls

## Data Availability

No new datasets were created that are posted.

## Supplementary Materials

The following supporting information can be downloaded at: https://www.mdpi.com/article/10.3390/cancers16213620/s1, Figure S1: Western blot (entire) stained for QSOX1 and for GAPDH. Densitometric intensities are shown below the blots. Video S1: *SuperScratch* Assay T98G/pLKO.1; Video S2: *SuperScratch* Assay T98G/sh86; Video S3: Mixed *SuperScratch* Assay 25% T98G/sh86/DiI with 75% T98G/sh86; Video S4: Mixed *SuperScratch* Assay 25% T98G/sh86/DiI with 75% T98G/sh86 phase; Video S5: Mixed *SuperScratch* Assay 25% T98G/sh86/DiI with 75% T98G/pLKO.1; Video S6: Mixed *SuperScratch* Assay 25% T98G/sh86/DiI with 75% T98G/pLKO.1 phase.
