# Supplementary figures and images for: QSOX1 Modulates Glioblastoma Cell Proliferation and Migration In Vitro and Invasion In Vivo
Source: Cancers (Basel). 2024 Oct 26;16(21):3620. doi: 10.3390/cancers16213620 (PMC11545231; doi:10.3390/cancers16213620)

Supplemental Figure S1

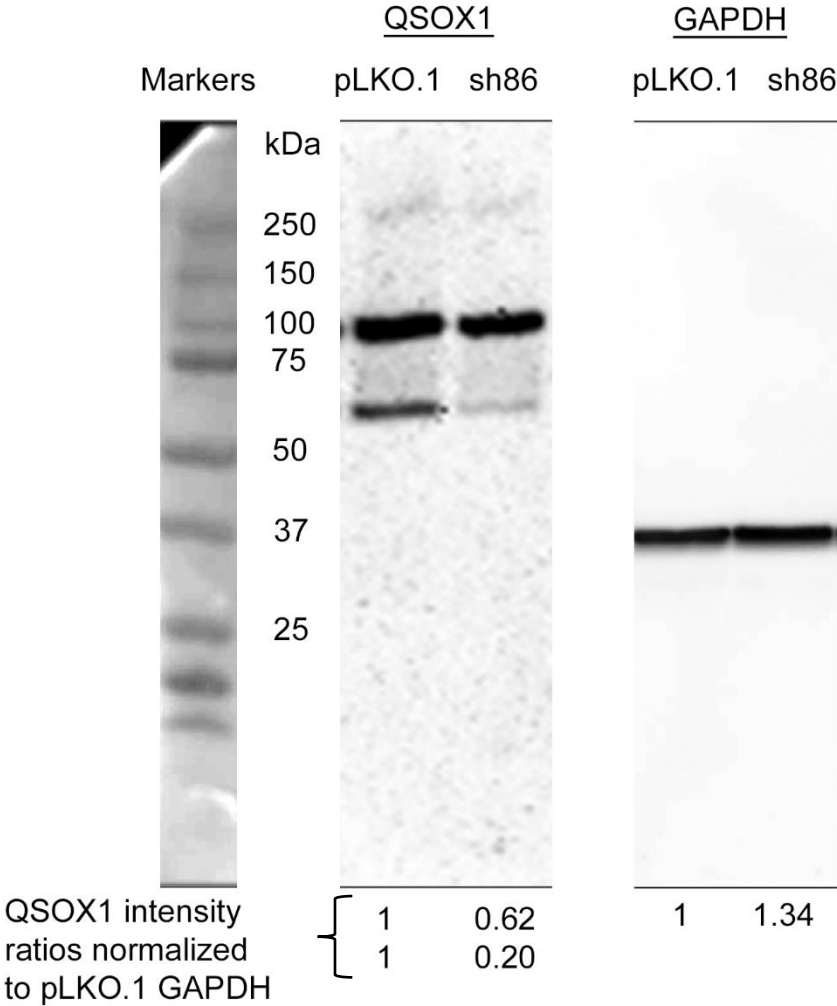

Supplement: Supplementary file 1 [file cancers-16-03620-s001.zip › Supplemental Figure S1.pdf]
